# All-trans retinoic acid and dexamethasone regulate phagocytosis-related gene expression and enhance dead cell uptake in C2C12 myoblast cells

**DOI:** 10.1038/s41598-023-48492-9

**Published:** 2023-11-28

**Authors:** Maysaa Adil Ali, Éva Garabuczi, Nastaran Tarban, Zsolt Sarang

**Affiliations:** 1 Faculty of Medicine, Doctoral School of Molecular Cell and Immune Biology, University of Debrecen, Debrecen, Hungary; 2 Department of Integrative Health Science, Faculty of Health Science, Institute of Health Science, University of Debrecen, Debrecen, Hungary; 3 Department of Biochemistry and Molecular Biology, Faculty of Medicine, University of Debrecen, Debrecen, Hungary

**Keywords:** Cell biology, Molecular biology, Differentiation

## Abstract

Extensive mechanical stress frequently causes micro-traumas in skeletal muscle, followed by a regeneration period. The effective removal of dead myofibers is a prerequisite for proper regeneration, and several cell types, including professional phagocytes, were reported to be active in this process. Myoblasts express several molecules of the phagocytic machinery, such as BAI1, stabilin-2, and TAM (Tyro3, Axl, Mertk) tyrosine kinase receptors, but these molecules were reported to serve primarily cell fusion and survival, and their role in the phagocytosis was not investigated. Therefore, we aimed to investigate the in vitro phagocytic capacity of the C2C12 mouse myoblast cell line. RNA sequencing data were analyzed to determine the level and changes of phagocytosis-related gene expression during the differentiation process of C2C12 cells. To study the phagocytic capacity of myoblasts and the effect of dexamethasone, all-trans retinoic acid, hemin, and TAM kinase inhibitor treatments on phagocytosis, C2C12 cells were fed dead thymocytes, and their phagocytic capacity was determined by flow cytometry. The effect of dexamethasone and all-trans retinoic acid on phagocytosis-related gene expression was determined by quantitative PCR. Both undifferentiated and differentiated cells engulfed dead cells being the undifferentiated cells more effective. In line with this, we observed that the expression of several phagocytosis-related genes was downregulated during the differentiation process. The phagocytosis could be increased by dexamethasone and all-trans retinoic acid and decreased by hemin and TAM kinase inhibitor treatments. Our results indicate that myoblasts not only express phagocytic machinery genes but are capable of efficient dead cell clearance as well, and this is regulated similarly, as reported in professional phagocytes.

## Introduction

Phagocytosis of dead cells by macrophages starts in vivo by locating the prey with the help of 'find me' signals released from the dying cells^1^. Next, macrophages recognize the dead cells' characteristic cell surface modifications with their phagocytic receptors. The most well-known modification is the appearance of phosphatidylserine (PS), a crucial ‘eat me’ signal, on the surface of dying cells^2^. Several receptors on the surface of macrophages directly recognize PS, such as stabilin-2 (Stab2), Brain angiogenesis inhibitor 1 (Bai1), or scavenger receptor class BI (Scarb1), while others, like TAM (Tyro3, Axl, Mer) receptor tyrosine kinases, use bridging molecules to interact with it^3^. Milk fat globule-EGF-factor 8 (MFG-E8), Growth arrest-specific protein 6 (Gas6), and Protein S (Pros1) are examples of these bridging molecules binding to integrin receptors β3 and β5 and TAM receptors, respectively^4,5^. Integrin receptors also need coreceptors such as Tim4^6^, transglutaminase 2 (Tgm2)^7^, or CD36^8^ in addition to bridging molecules for appropriate phagocytic activity. Various efferocytosis receptor combinations are produced by phagocytes, in different organs, but they all come and work together at the phagocytic synapse to mediate tethering and generate enough engulfment signals after macrophages connect to the apoptotic cells^9^. Previously, the glucocorticoid receptor (GR) and retinoic acid receptors (RARs) nuclear receptors were shown to increase efferocytosis in macrophages by promoting the transcription of key phagocytic machinery genes^10–12^.

Myoblasts are precursor cells that give rise to muscle fibers. They are found in skeletal and cardiac muscle and are responsible for muscle development, growth, and repair^13^. During their differentiation, myoblasts fuse and form multinucleated myotubes that eventually form muscle fibers. Myoblast fusion is initiated by the alignment of myoblast and myotube membranes, followed by rearrangements of the actin cytoskeleton at the contact sites, and then membrane fusion^14^. Like dead cell phagocytosis, this process depends on PS exposure on the fusing cell surface^15^. Besides the muscle-specific fusion proteins myomaker and myomerger^16^, myoblasts and myotubes also express several PS-recognizing phagocytic receptors and bridging molecules such as the TAM family members, integrin β1, β3 and β5, Bai1, Stab2, MFG-E8, and Gas6 that are traditionally expressed by macrophages, dendritic cells, and other professional phagocytes^3^. In myoblasts, these molecules were shown to participate in cell adhesion, fusion, and survival^14,17–19^. Although living C2C12 cells were found to engulf apoptotic C2C12 cells, their phagocytic capacity was not investigated in detail^17^. Previously, RAR and GR ligation were reported to regulate key aspects of myoblast functions. All-trans retinoic acid (ATRA) treatment suppressed myogenic differentiation and myotube formation in human primary myoblasts^20^, but it was shown to be required for myogenesis in zebrafish^21^. GR ligation promotes muscle repair and regeneration by increasing the kinesin-1 motor activity, which is needed for the expression of muscle myosin heavy chain 1/2, and also for efficient myoblast fusion and the formation of polarized myotubes^22^ but the effect of retinoid- and glucocorticoid receptor ligation on dead cell phagocytosis in myoblasts has not been investigated yet. In this study, we tested the hypothesis that myoblast phagocytosis could be similarly regulated by RAR or GR ligands and hemin as in macrophages.

## Methods

All reagents were obtained from Merck, (Darmstadt, Germany) except when indicated otherwise.

### Gene expression data processing

Gene expression data, generated from GPL17021 Illumina HiSeq 2500 (Mus musculus), corresponding to three undifferentiated three 4-day differentiated C2C12 cell samples, was retrieved from the public Gene Expression Omnibus (GEO) repository (GEO series GSE220249). For a detailed description of the sample preparation and sequencing protocol, see the original paper by Zhang et al.^23^. FPKM (Fragments per kilobase of transcript per million mapped fragments) values of samples were compared using two-tailed t-test. The equal variance of the groups was tested by F-test. Data were imported in Microsoft Access and cross-referenced to a list of 241 phagocytosis-related genes (based on Gene Ontology term ID 0006909, phagocytosis). A list of differently expressed genes (DEGs) was generated by removing non-significantly changed transcripts based on a 0.05 p-value cut-off (Student’s t-test) and a 1.5-fold fold change (FC) cut-off value. For the visualization, FPKM values were log2 transformed, and the Z-score was calculated for every DEG and plotted using the http://www.heatmapper.ca/expression/ web tool.

### C2C12 cell culture and differentiation

Murine myoblast C2C12 cells were obtained from ATCC (CRL-1772) and were maintained according to the company’s instructions. In brief, cells were cultured in Dulbecco’s modified Eagle’s medium (DMEM) supplemented with 20% fetal bovine serum (FBS), 100 U/ml penicillin, and 100 μg/ml streptomycin (growth medium) at 37 °C in 5% CO2 and 95% air at 100% humidity. The absence of mycoplasma was tested using PCR Mycoplasma Test Kit I/C (PromoCell, Heidelberg, Germany). Cells were plated into 24-, 48-, or 96-well plates at a density of 3500 cells/cm^2^. For the 6-day differentiation period, the cells were plated on a collagen-coated surface and cultured in DMEM medium containing 2% FBS and 1% ITS (insulin, transferrin, sodium selenite) replaced every 2nd day with a fresh one. In some experiments, C2C12 cells were pre-treated with dexamethasone (1 or 10 μM) or ATRA (30 nM) or hemin (5 μM) for 24 h or BMS-777607 TAM kinase inhibitor (1 or 10 μM) for 30 min prior to the phagocytosis experiments or RNA collection.

### Immunostaining

For MYHC4 staining, 6-day differentiated C2C12 cells were fixed with ice-cold methanol for 15 min at 4 °C, washed three times with PBS, and then blocked with PBS/5% FBS/0.3% Triton X-100 for 60 min at room temperature. Alexa Fluor 488 conjugated anti-Myosin heavy chain 4 (Thermo Fisher Scientific) was added at 1:100 dilution in PBS/1% bovine serum albumin/0.3% Triton X-100 for 12 h at 4 °C. After staining, cells were rinsed three times in PBS and counterstained with NucBlue for DNA staining. Pictures were taken on a fluorescent microscope (FLoid™ Cell Imaging Station).

### Gene expression analysis

Total RNA from C2C12 cells was isolated with TRIzol (Invitrogen, Carlsbad, CA, USA) reagent according to the manufacturer’s instructions. Total RNA was reverse transcribed into cDNA using a High-Capacity cDNA Reverse Transcription Kit (Thermo Fisher Scientific, Waltham, MA, USA) according to the manufacturer’s instructions. Quantitative reverse transcription polymerase chain reaction (RT–qPCR) was carried out in triplicate using pre-designed PCR assays (Thermo Fisher Scientific) on a Roche LightCycler LC 480 real-time PCR instrument. Relative mRNA levels were calculated using the comparative CT method and were normalized to Casein Kinase 2 Alpha 2 (Csnk2a2) mRNA. Catalog numbers of the TaqMan assays (Thermo Fisher Scientific) used were the following: Csnk2a2 Mm00441242_m1, MFG-E8 Mm00500549_m1, TGM2 Mm00436979_m1, CD36 Mm00432403_m1, Pros1 Mm01343426_m1, Tyro3 Mm00444547_m1, MERTK Mm00437221_m1, AXL Mm00500549_m1, Gas6 Mm00490378_m1, UCP2 Mm00627599_m1, Stab2 Mm00454684_m1, HMOX1 Mm00516005_m1, MyoD1 Mm00440387_m1, Myog Mm00446194_m1, and Myh4 Mm01332541_m1.

### Generation of apoptotic and necrotic cells

Thymi from 4-week-old C57BL/6 mice were collected and placed in 10 ml physiological saline solution. Thymocytes were separated by gently pushing the thymus through a metal mesh filter. The cells were then filtered through a 41 μm filter and centrifuged for 8 min at 200 g. The pellet was resuspended in 10 ml RPMI 1640 media supplemented with 2 mM glutamine, 100 U/ml penicillin, and 100 μg/ml streptomycin in the absence of FBS and incubated for 20 h (10^7^ cells/ml). This treatment results in approximately 80% annexin V positive cells^24^. Apoptotic thymocytes were then stained by 0.5 μM CellTracker™ Deep Red dye (Invitrogen) at 37 °C for 30 min in the absence of FBS to be used as target cells to detect efferocytosis by C2C12 cells. For necrosis induction, cells were first labeled with 0.5 μM CellTracker™ Deep Red dye at 37 °C for 30 min in the absence of FBS and then heated to 65 °C for 10 min.

### In vitro phagocytosis assay

Proliferating or differentiating C2C12 cells were grown in 48-well plates as described above. Deep red-stained apoptotic or necrotic thymocytes were added to the C2C12 cells in a 5:1 (dead cells/C2C12 cells) ratio on the day of the flow cytometric measurement for 30 or 60 min. After co-culture, non-engulfed target cells were washed away extensively with phosphate-buffered saline, and C2C12 cells were detached by trypsinization at 37 °C for 15 min and filtered through a 100 μm filter before the measurement to eliminate cell clots. The percentage and the mean fluorescence of engulfing cells were determined on a Becton Dickinson FACSCalibur™ flow cytometer (Becton Dickinson Company, Franklin Lakes, NJ, USA). The obtained data was analyzed using Flowing Software v2.5.1 (Turku Centre for Biotechnology, University of Turku, Finland). To visualize thymocyte phagocytosis, 0.5 μM CellTracker Deep Red dye-stained C2C12 cells were incubated with carboxyfluorescein diacetate succinimidyl ester (CFDA-SE; Thermo Fisher) stained apoptotic thymocytes at 37 °C for 60 min. Cells were rinsed in PBS and fixed with 2% paraformaldehyde. Pictures were taken on a fluorescent microscope (FLoid™ Cell Imaging Station).

### Cell viability determination

To determine the cell numbers, C2C12 cells were seeded onto 96-well plates in growth medium and treated with DMSO or 5 μM hemin for 24 h. Non-toxic, cell-permeable PrestoBlue (Thermo Fisher Scientific) dye was added to the wells (1:10), and fluoresce was measured at the indicated time points on a Synergy™ H1 microplate reader at 560/590 nm (excitation/emission).

### Statistical analysis

All the data are representative of at least three independent experiments, and all data are expressed as mean ± SD. Statistical analysis was performed using two-tailed, unpaired Student's t-test and ANOVA with post-hoc Tukey HSD test. The equal variance of the samples was tested by F-test. * and # indicate statistically significant differences at *p* < 0.05.

## Results

### The myogenic differentiation impairs the phagocytic capacity of myoblasts

**Figure 1:**
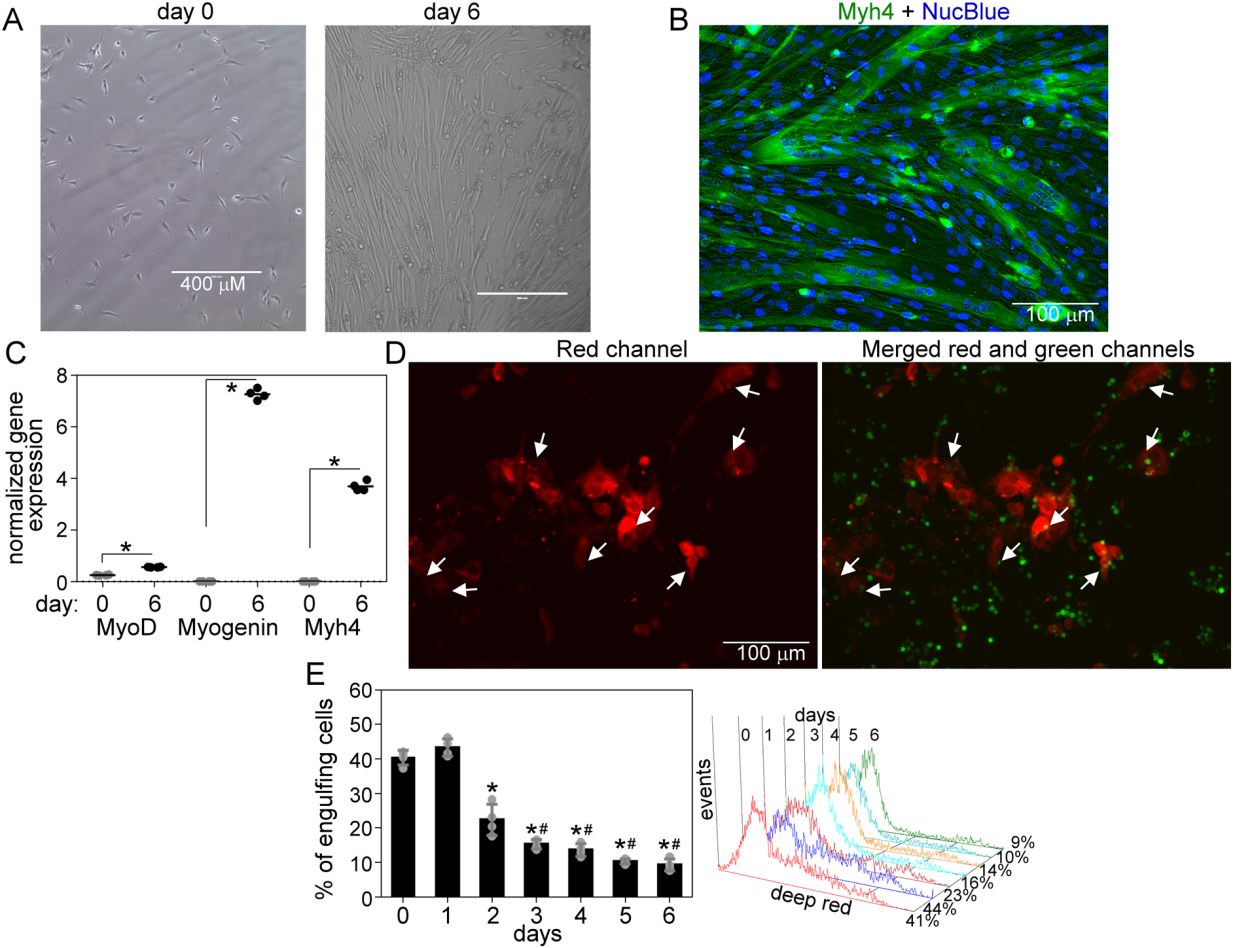
Myogenic differentiation impairs the phagocytic capacity of myoblasts. (**A**) Representative light microscopic picture of proliferating and 6-day differentiated C2C12 myoblasts. (**B**) Representative fluorescent picture of 6-day differentiated C2C12 cells forming multinucleated myotubes. (**C**) The expression level of three myogenic differentiation markers in proliferating and 6-day differentiated C2C12 cells was measured by RT-qPCR. (**D**) Representative fluorescent pictures of proliferating C2C12 cells (red) phagocytosing apoptotic thymocytes (green). The arrows indicate locations with internalized thymocytes. (**E**) Percentage of apoptotic thymocyte engulfing undifferentiated and differentiated C2C12 determined by flow cytometry. The right panel shows representative flow cytometry histograms of phagocytosing myoblasts. Asterisks denote statistically significant differences from day 0, and # denotes statistically significant differences from day 2 (*p* < 0.05, ANOVA-test). n = 4.

In our experiments, we used the C2C12 myoblast cells established from normal adult C3H mouse leg muscle. Upon serum withdrawal, these cells are capable of differentiating into myotubes in vitro in six days (Fig. 1A,B, and C). To show that C2C12 cells are capable of internalizing dead cells, labeled myoblast and apoptotic thymocytes were mixed, and fluorescent pictures were taken. The phagocytosed thymocytes appear as black holes in the red C2C12 cells (Fig. 1D). To determine the phagocytic capacity of proliferating and differentiating myoblasts, C2C12 cells were fed apoptotic thymocytes for 1 h, and the percentage of engulfing cells was measured by flow cytometry. Compared to proliferating cells, the phagocytic capacity significantly decreased on the second day of differentiation and continued to decline until the sixth day (Fig. 1E).

### Myogenic differentiation impacts the expression of phagocytosis-related genes in myoblasts

**Figure 2:**
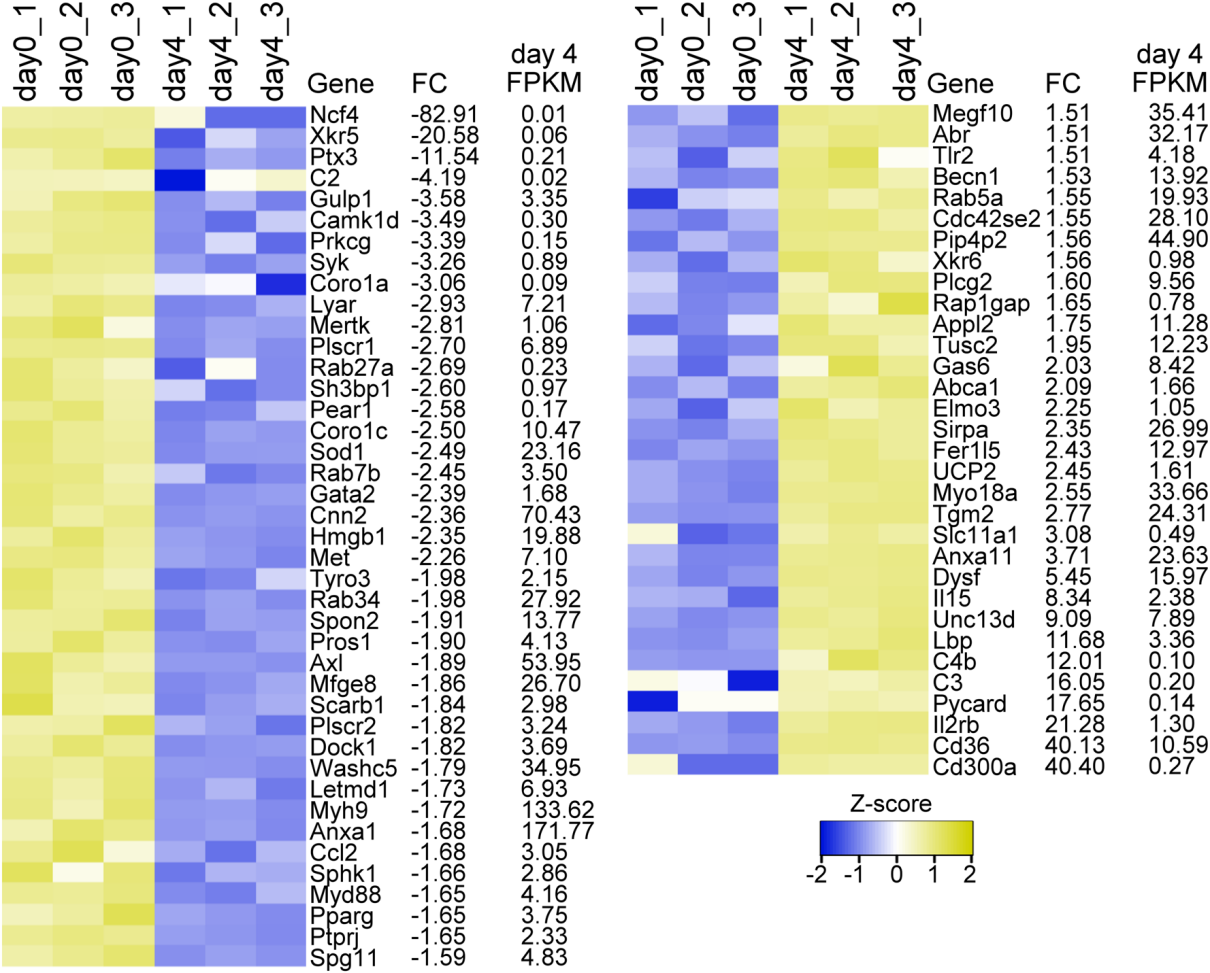
Myogenic differentiation impacts the gene expression profile in myoblasts. The heat map displays the Z-score calculated from the log2 transformed FPKM values of 73 phagocytosis-associated DEGs between proliferating and 4-day differentiated C2C12 cells. The significance of FCs was tested by Student’s t-test, *p* < 0.05. The individual FC values and day 4 FPKM values, representing the expression level of the transcripts on the 4th day of differentiation are shown to the right of the heat maps. n = 3.

**Figure 3:**
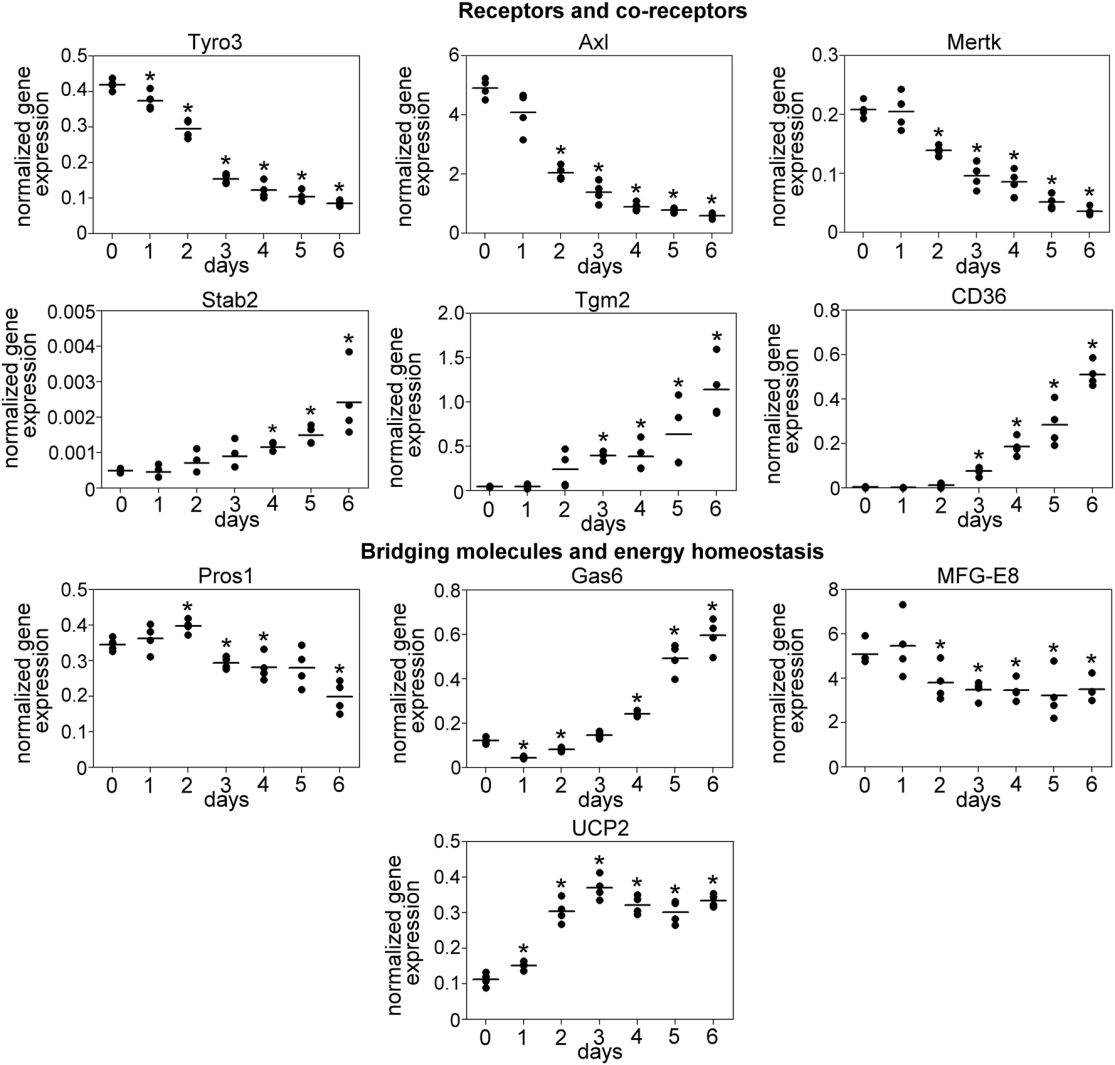
Differentiation alters the expression of phagocytosis-associated genes in C2C12 cells. The mRNA expression level of 10 phagocytosis-associated genes was validated by RT-qPCR. Asterisks denote statistically significant differences from day 0 (*p* < 0.05, Student’s t-test). n = 4.

Myogenic differentiation has a profound impact on the gene expression pattern of myoblast. To investigate the possibility that the reduced phagocytosis is the consequence of altered expression of phagocytosis-associated genes, we downloaded gene expression data of proliferating and 4-day differentiated C2C12 cells from the GEO database and cross-referenced it into a list containing 241 phagocytosis-associated genes (based on the Gene Ontology term ID 0006909). From the 178 expressed list members, we found 73 DEGs among them, 41 were downregulated (day 4 mean and median FPKM values were 16.2 and 3.49, respectively), and 32 were upregulated (day 4 mean and median FPKM values were 12.2 and 8.98, respectively) on 4th day of the differentiation (Fig. 2). Using quantitative PCR, we validated the mRNA expression of the downregulated MFG-E8, the TAM family members and their ligands, Pros1, and the upregulated Gas6, Tgm2, UCP2, and CD36 that was the second highest upregulated transcript detected by the RNA sequencing. Stab2 expression increased in the differentiated cells (FC = 2.687), but it did not reach the significance threshold (*p* = 0.0701) therefore, we also measured its expression by RT-qPCR. Starting from the 4th day, Stab2 expression was significantly upregulated during the differentiation (Fig. 3).

### Dexamethasone treatment enhances the apoptotic cell uptake in myoblasts and regulates the expression of phagocytosis-related genes

**Figure 4:**
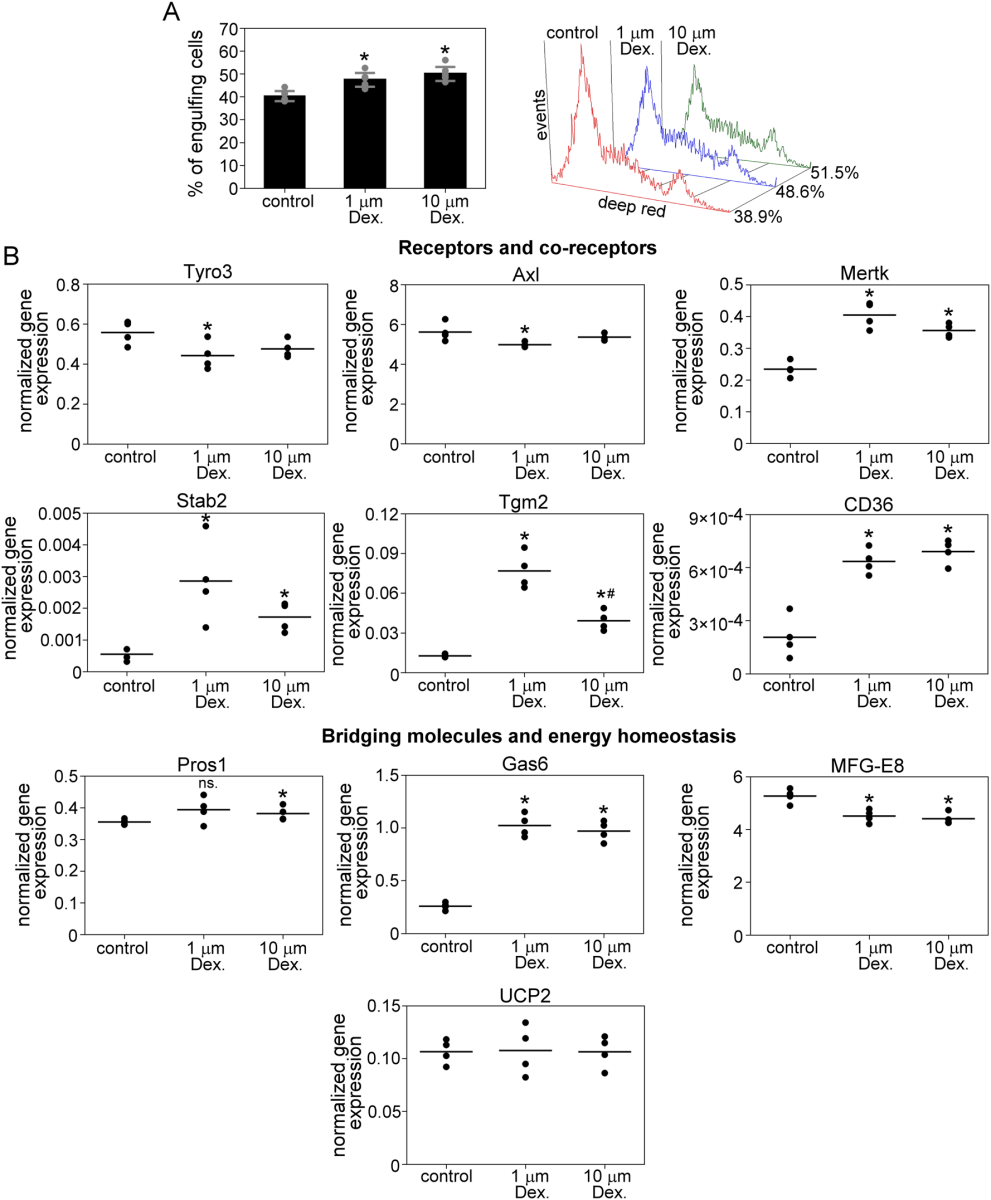
Dexamethasone treatment enhances apoptotic cell uptake in myoblasts and regulates the expression of phagocytosis-related genes. (**A**) Percentage of apoptotic thymocyte engulfing DMSO-treated control and dexamethasone-treated (1 or 10 μM for 24 h) C2C12 cells determined by flow cytometry. The right panel shows representative flow cytometry histograms of phagocytosing myoblasts. (**B**) The expression level of 10 phagocytosis-associated genes in DMSO control and dexamethasone-treated C2C12 was measured by RT-qPCR. Asterisks denote statistically significant differences from day 0, and # denotes statistically significant differences from 1 μM treated cells (*p* < 0.05, ANOVA-test). n = at least 4. ns: non-significant.

GR ligation was shown to enhance macrophage phagocytic capacity^25,26^, and according to RNA sequencing data, C2C12 cells express GR. Therefore, we decided to investigate its impact on phagocytosis in C2C12 cells as well. We found that 24 h of dexamethasone treatment significantly increased the apoptotic cell phagocytosis in myoblasts (Fig. 4A). Parallel with this, the mRNA expression of Stab2, Mertk, Gas6, Tgm2, and CD36 was significantly upregulated, while that of Tyro3, Axl, and MFG-E8 was downregulated by dexamethasone (Fig. 4B).

### ATRA treatment enhances the apoptotic cell uptake in myoblasts and regulates the expression of phagocytosis-related genes

**Figure 5:**
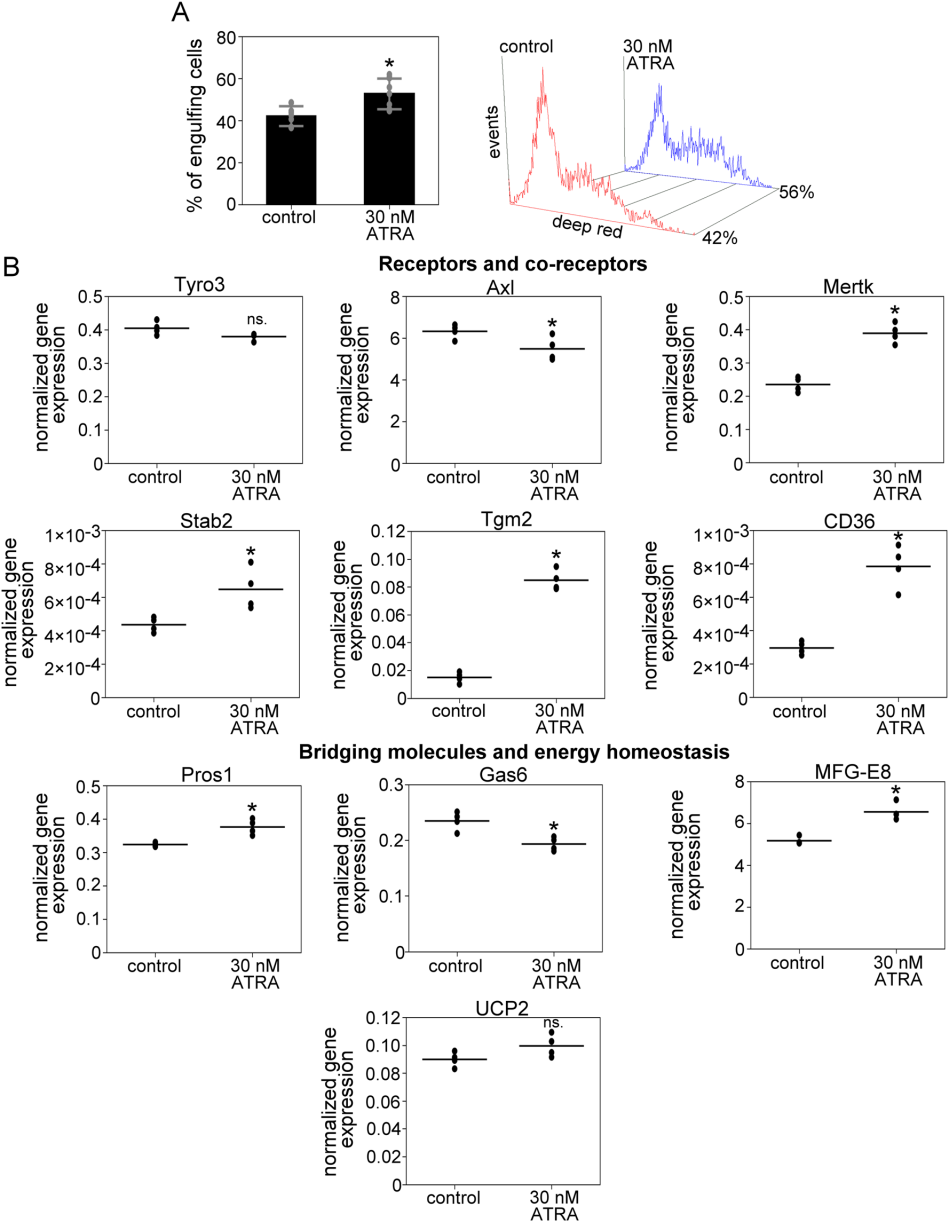
ATRA treatment enhances apoptotic cell uptake in myoblasts and regulates the expression of phagocytosis-related genes. (**A**) Percentage of apoptotic thymocyte engulfing DMSO-treated control and ATRA-treated (30 nM for 24 h) C2C12 cells determined by flow cytometry. The right panel shows representative flow cytometry histograms of phagocytosing myoblasts. (**B**) The expression level of 10 phagocytosis-associated genes in DMSO-treated control and ATRA-treated C2C12 was measured by RT-qPCR. Asterisks denote statistically significant differences from day 0 (*p* < 0.05, Student’s t-test). n = at least 4. ns: non-significant.

Since previously we demonstrated that retinoids enhance the phagocytic capacity of macrophages^27,28^, and based on the RNA sequencing data, C2C12 cells express all three RAR isoforms, we decided to test its effect on phagocytosis in C2C12 cells. We found that 24 h of ATRA treatment significantly increased the apoptotic cell phagocytosis in myoblasts (Fig. 5A). Parallel with this, the mRNA expressions of Stab2, Mertk, Pros1, MFG-E8, Tgm2, and CD36 were significantly upregulated, while that of Axl and Gas6 was downregulated by ATRA (Fig. 5B).

### C2C12 cells partially use the TAM receptor kinases to engulf both apoptotic and necrotic thymocytes with equal efficiency

**Figure 6:**
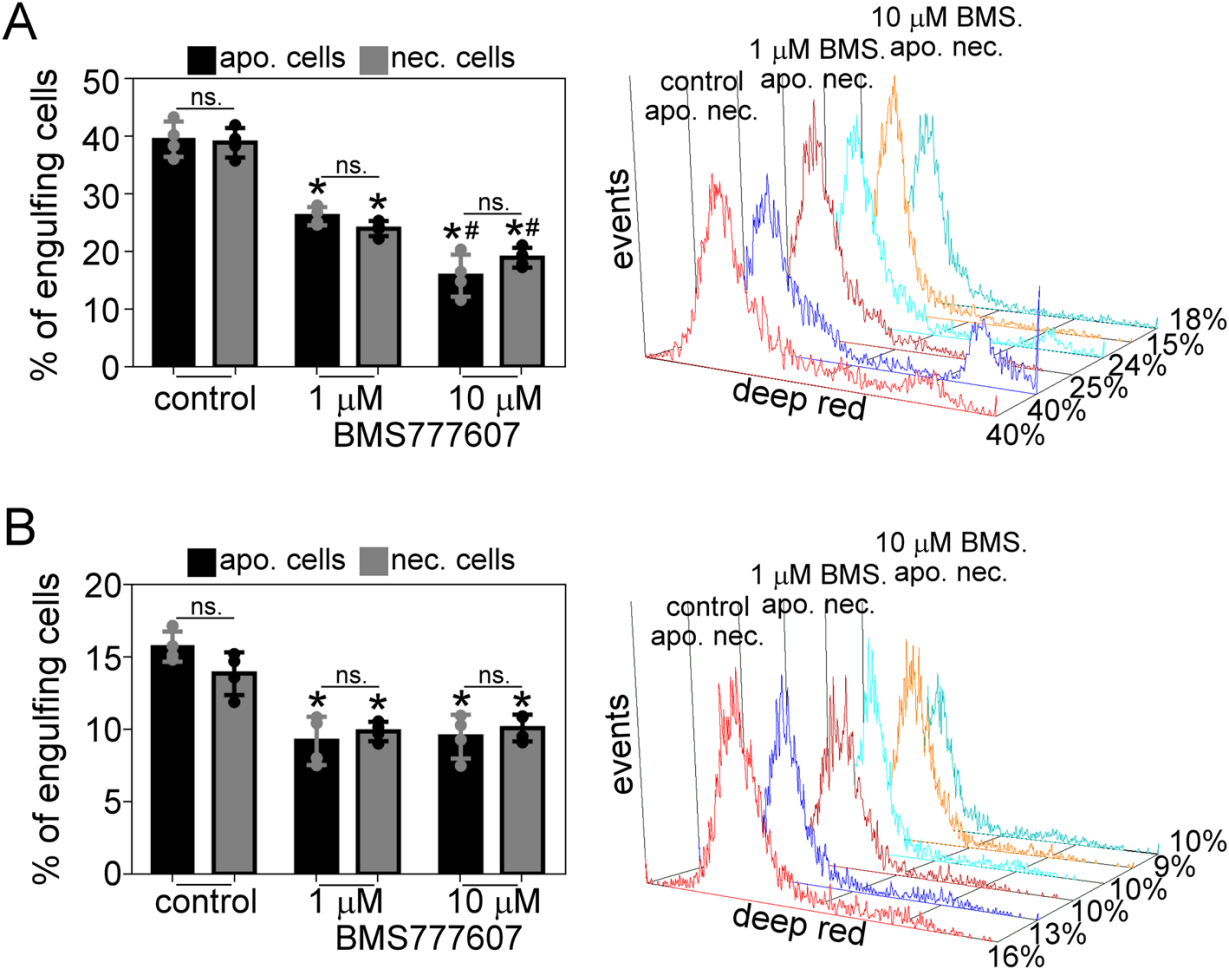
C2C12 cells partially use the TAM receptor kinases to engulf both apoptotic and necrotic thymocytes with equal efficiency. (**A**) Proliferating and (**B**) 6-day differentiated C2C12 cells were fed apoptotic or necrotic thymocytes in the absence or presence of BMS-777607 TAM kinase inhibitor (1 or 10 μM for 24 h), and the percentage of engulfing cells was determined by flow cytometry. The right panel shows representative histograms of phagocytosing myoblasts. Asterisks denote statistically significant differences from DMSO control, and # denotes statistically significant differences from the respective 1 μM treated cells (*p* < 0.05, ANOVA-test). n = 4. ns: non-significant.

Since previously we have shown that macrophages engulf apoptotic and necrotic thymocytes through similar PS-dependent mechanisms^29^, we decided to compare the efficiency of apoptotic and necrotic cell phagocytosis in proliferating and 6-day differentiated myoblast cells. We found that both undifferentiated and differentiated C2C12 engulfed dead thymocytes regardless of the cell death mechanism (Fig. 6A,B), being the undifferentiated C2C12 cells more efficient. Since C2C12 cells express the TAM receptors and their mRNA expression decreases during the myogenic differentiation, we also tested whether the apoptotic and necrotic cell uptake requires the TAM receptors. The TAM kinase inhibitor BMS-777607 significantly decreased both apoptotic and necrotic cell phagocytosis. In agreement with the decreased TAM receptor expression in the differentiated cells, this inhibition was more pronounced in the case of the undifferentiated cells as the phagocytosis inhibition by 10 μM BMS-777607 treatment was 60% and 51% in the proliferating cells and 39% and 22% in the differentiated cells for apoptotic and necrotic cell uptake, respectively (Fig. 6A,B).

### Hemin inhibits the phagocytic capacity and upregulates heme oxygenase-1 (HMOX1) expression in C2C12 myoblast cells

**Figure 7:**
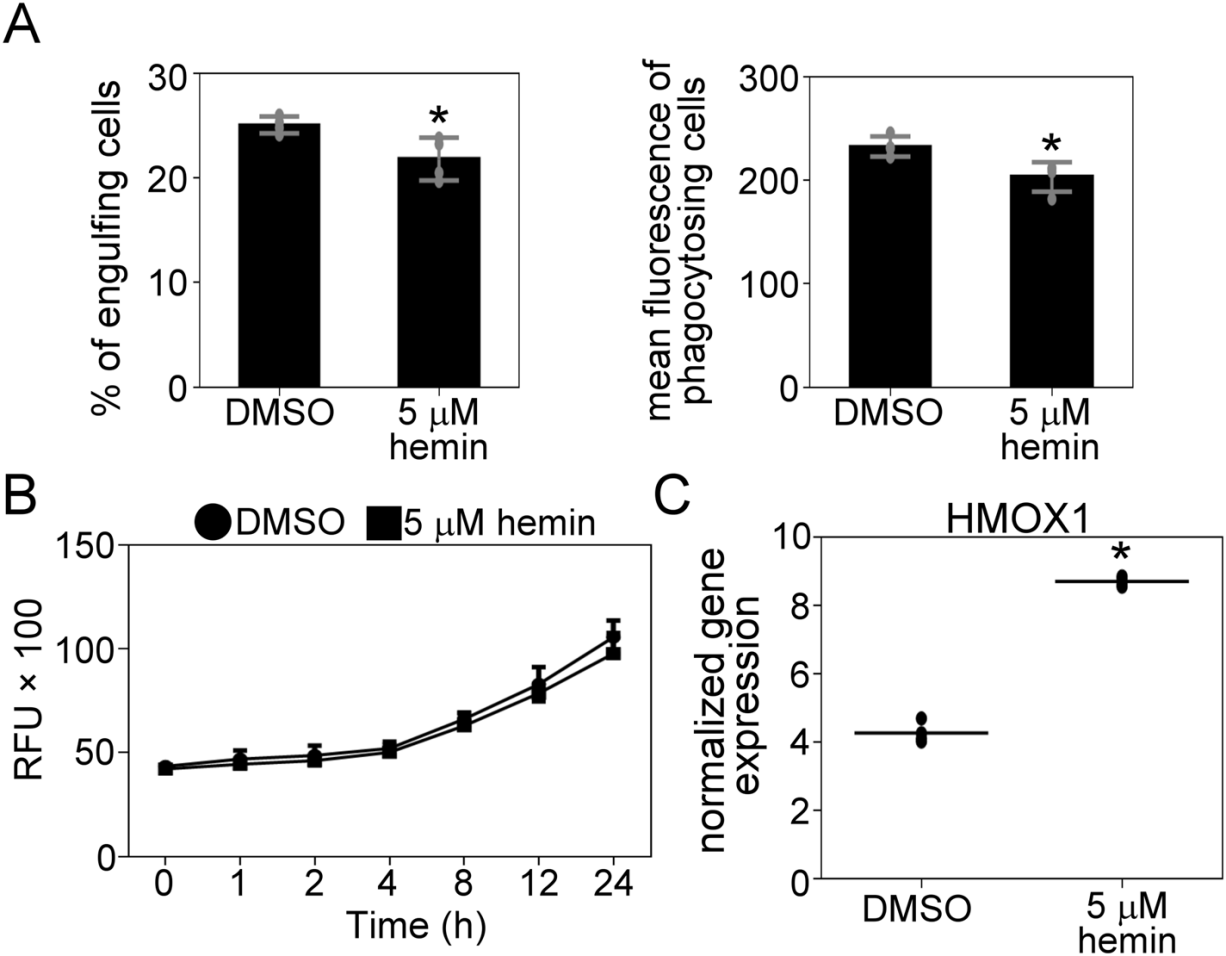
Hemin inhibits the phagocytic capacity and upregulates heme oxygenase-1 expression in C2C12 myoblast cells. (**A**) Proliferating DMSO or hemin (5 μM for 24 h) treated C2C12 cells were fed apoptotic thymocytes for 30 min, and the percentage and mean fluorescence of the engulfing cells were determined by flow cytometry. (**B**) The effect of 5 μM hemin treatment of the C2C12 cell proliferation was tested by PrestoBlue staining. (**C**) Expression levels of HMOX1 in DMSO or hemin-treated C2C12 cells determined by RT-qPCR. Asterisks denote statistically significant differences from day 0 (*p* < 0.05, Student’s t-test). n = 4.

Previously, it was reported that heme inhibits the uptake of human and bacterial cells by macrophages^30,31^, therefore, we sought to investigate whether myoblasts also behave in the same manner. As the free heme is not stable and ferrous Fe^2+^ iron inside the heme quickly oxidizes to ferric Fe^3+^, we pretreated the C2C12 cells with 5 μM Fe^3+^-containing hemin for 24 h and tested their phagocytic capacity. As shown in Fig. 7A and B, 24 h of 5 μM hemin treatment was not toxic for cells but inhibited their phagocytosis capacity. We also measured the mRNA expression of the HMOX1 gene, coding for the first enzyme heme degradation pathway. Similarly to what was reported in macrophages, its expression increased in response to hemin overload (Fig. 7C).

## Discussion

The proper clearance of dead cells is required for the maintenance of tissue homeostasis and proper regeneration following injury^32^. Professional phagocytes are equipped with a battery of receptors to recognize, bind, and engulf dead cells^1^. Myoblasts and myotubes, including C2C12 cells, have been found to express a variety of phagocytic receptors such as scavenger receptors, mannose receptors, Stab2, Bai1, and TAM family members that are traditionally expressed by macrophages, dendritic cells, and other phagocytic cells. The expression of these receptors in myoblasts and myotubes suggests that these cells have the potential for efficient dead-cell engulfment. To investigate this possibility, we fed proliferating C2C12 myoblast cells with apoptotic thymocytes and found that within the one-hour phagocytosis period, more than 40% of the cells engulfed at least one dead cell. This result is comparable to what we detected previously in bone marrow-derived macrophages^29^. We also measured the phagocytic capacity of differentiating cells and found that it gradually decreased during the differentiation period. Differentiation profoundly alters the gene expression profile of cells. Recently, using a robust rank aggregation method, 3140 DEGs were identified in differentiating C2C12 cells^33^. In our analysis, among others, we found decreased mRNA expression of the Scarb1 receptor, TAM receptors Tyro3, Axl, and Mertk and their ligand Pros1, and the bridging molecule MFG-E8 that can contribute to the diminished phagocytic capacity of the differentiated cells. Previously, we have shown that in C2C12 cells, the Axl is the highest expressed TAM kinase whose expression could be detected on the protein level, and it is required for myoblast survival during the differentiation^18^. Notably, the phagocytosis could be inhibited not only in proliferating but in differentiated C2C12 cells as well by pan-TAM kinase receptor inhibitor, indicating that even at the decreased expression level, Axl still mediates the efficient engulfment of dead cells. The scavenger receptor CD36 was the second highest upregulated DEG. Previously, its expression was shown to be increased in differentiating C2C12 cells, and knocking down its expression impaired myoblast differentiation and fusion^34^. CD36 with αvβ3 integrin binds the bridging molecule thrombospondin 1 and facilitates the apoptotic cell uptake in macrophages^35^. αvβ3 integrin with Tgm2 and MFG-E8 was also shown to be required for the proper phagocytosis in macrophages^7^. Recently, we have found that Tgm2 is needed for embryonic skeletal muscle development and muscle regeneration in mice, and its inhibition impairs C2C12 cell fusion^19^. As the disappearance of β3 integrin expression is a prerequisite for myoblast differentiation^36^ and differentiated C2C12 cells exhibited lower phagocytosis, the observed upregulation of CD36 and Tgm2 is likely required for myoblast differentiation and fusion rather than serving as a key phagocytic receptor in differentiated C2C12 cells. Similar to our results, Stab2 was reported to be upregulated in differentiating primary myoblasts and C2C12 cells, where it participates in cell fusion^37^. Given its low mRNA expression level compared to the other detected and downregulated phagocytic genes, although it is required for myoblast fusion, it might not have a determining role in phagocytosis. In macrophages, the continued clearance of apoptotic cells depends on the uncoupling protein 2 (UCP2) protein expression^38^. However, in C2C12 cells, UCP2 plays a role in inhibiting myogenic differentiation and promoting myoblast proliferation. The level of UCP2 transcript increased, but due to the miRNA-mediated translational block, its protein level decreased during the differentiation and was nearly undetectable in the 5-day differentiated cells^39^. Based on this, the role of UCP2 is fundamentally different in macrophages, and its upregulation at the mRNA level might not be relevant in the phagocytosis of differentiated C2C12 cells.

Previously, we have found that 24 h of ATRA treatment enhances the phagocytic capacity of bone marrow-derived macrophages and upregulates the expression of several phagocytosis-related genes, among others, Mertk, Stab2, and Tgm2^27^ therefore, we aimed to investigate its effect on myoblast cells as well. Similarly to macrophages, ATRA treatment enhanced the phagocytic capacity and increased the expression of these and, additionally, CD36, MFG-E8, and Pros1 genes at the mRNA level in C2C12 cells. As CD36 and Tgm2 and its binding partner MFG-E8 form PS-binding receptor complexes with integrin β3, we can hypothesize that they can be partially responsible for the increased phagocytosis of ATRA-treated undifferentiated C2C12 myoblasts. Others and we have shown that glucocorticoid receptor activation induces the expression of several phagocytic genes and results in enhanced dead cell clearance in macrophages^25,26^. Dexamethasone upregulated the expression Stab2, Mertk, Gas6, Tgm2, and CD36 at the mRNA level and increased the phagocytic capacity of C2C12 cells, as well.

Mertk expression is regulated by a GR response element in its promoter and contributes to dexamethasone-induced phagocytosis in macrophages^25^. Its mRNA level increased in response to glucocorticoid treatment in C2C12 cells as well, suggesting that it may contribute to the increased phagocytosis not only in macrophages but also in myoblast cells. According to the RNA sequencing analysis, Axl was the fourth highest expressed gene among the 73 DEG in the undifferentiated state with an average FPKM value of 101.98 (the mean and median DEG FPKM values in the undifferentiated cells were 20.22 and 5.48, respectively). Although its expression decreased moderately after dexamethasone and ATRA treatments, given its robust expression and the fact that TAM kinase inhibitor lowered the phagocytic capacity of the differentiated C2C12 cells with low TAM receptor expression, it might still contribute to the phagocytic capacity of these cells. As a failure of dead cell clearance results in improper muscle regeneration^18^, the dexamethasone-induced enhanced myoblast phagocytosis might contribute to the beneficial effect of glucocorticoid treatment on muscle repair^22^ due to the upregulation of phagocytosis-related fusion machinery gene expression, leading to enhanced dead-cell phagocytosis.

Similarly to apoptotic cells, the uptake of necrotic cells also depends on cell surface PS exposure^40,41^ and decreases by TAM receptor inhibition^29^ in macrophages. In our experiments, apoptotic and necrotic cells were engulfed at the same efficiency, indicating that myoblasts have no preference for either of them, and their uptake was inhibited in the presence of the TAM kinase inhibitor, similarly to macrophages. The more pronounced inhibitory effect of BMS-777607 on the proliferating cells might be attributed to the downregulation of TAM kinase expression in the differentiated cells, resulting in less TAM-dependent phagocytosis.

During tissue damage, red blood cells lyse at the injury site and release intracellular hemoglobin that releases heme. The ferrous iron in heme quickly oxidizes to ferric forming hemin. Due to their hydrophobic nature, heme and hemin are incorporated into biological membranes and cause lipid peroxidation and cell death. To prevent this harmful scenario, macrophages clear the hemoglobin-haptoglobin complex via their CD163 receptor and hemin with CD36 receptor and upregulate the expression of HMOX1, which degrades the porphyrin ring^42,43^. The heme degradation products biliverdin and bilirubin have antioxidant and anti-inflammatory effects, contributing to tissue repair after injury^44^. In addition, the exposure of macrophages to free heme or hemin decreased bacterial and apoptotic cell phagocytosis^30,31^. Hemin was shown to lower phagocytosis by disrupting the actin cytoskeletal phagocytic machinery and by facilitating the removal of CD36 receptors from the cell membrane^31,43^. According to the RNA sequencing and qPCR data, the C2C12 myoblasts also express CD163 and CD36 and upregulate the HMOX1 gene in response to hemin exposure, which might confer protection against the toxic effect of hemoglobin- and myoglobin-derived heme and hemin and limit the inflammation following muscle injury. Moreover, in our experiments, similarly to macrophages, hemin also decreased the uptake of apoptotic thymocytes by C2C12 cells, although how this effect is mediated remains to be elucidated.

## Conclusion

Gene expression analysis and phagocytosis assays demonstrated that C2C12 myoblast cells share several characteristics of professional phagocytes reported previously in the literature in terms of phagocytic machinery gene expression and regulation of dead cell clearance. We have shown that differentiation, TAM receptor kinase inhibition, and hemin treatment decrease, while ATRA and dexamethasone increase the dead cell uptake in myoblast cells. Our findings indicate that by engulfing dead cells and degrading harmful hemin, myoblasts might contribute to cell debris clearance, a prerequisite for proper muscle regeneration following injury.

The limitation of the study is that RNA sequencing and RT-qPCR measure the abundance of RNAs, and gene expression levels do not always reflect changes at the protein level, therefore, it needs to be later verified by protein-detecting methods such as Western blot and ELISA.

## Data Availability

RNA-sequencing GSE220249 data series is available on the GEO website. All raw data used to generate the figures are available upon request from the corresponding author.
